# Efficacy and safety of femoral nerve block for the positioning of femur fracture patients before a spinal block – A systematic review and meta-analysis

**DOI:** 10.1371/journal.pone.0216337

**Published:** 2019-05-02

**Authors:** Yuan-Pin Hsu, Chin-Wang Hsu, Karen Chia Wen Chu, Wen-Cheng Huang, Chyi-Huey Bai, Chun-Jen Huang, Sheng-Wei Cheng, Jin-Hua Chen, Chiehfeng Chen

**Affiliations:** 1 Emergency Department, Wan Fang Hospital, Taipei Medical University, Taipei, Taiwan; 2 Graduate Institute of Clinical Medicine, College of Medicine, Taipei Medical University, Taipei, Taiwan; 3 Department of Emergency, School of Medicine, College of Medicine, Taipei Medical University, Taipei, Taiwan; 4 Department of Public Health, School of Medicine, College of Medicine, Taipei Medical University, Taipei, Taiwan; 5 Department of Anesthesiology, Wan Fang Hospital, Taipei Medical University, Taipei, Taiwan; 6 Division of Gastroenterology, Department of Internal Medicine, Wan Fang Hospital, Taipei Medical University, Taipei, Taiwan; 7 Graduate Institute of Data Science, College of Management, Taipei Medical University, Taipei, Taiwan; 8 Biostatistics Center, College of Management, Taipei Medical University, Taipei, Taiwan; 9 Department of Medical Education and Research, Wan Fang Hospital, Taipei Medical University, Taipei, Taiwan; 10 Cochrane Taiwan, Taipei Medical University, Taipei, Taiwan; 11 Division of Plastic Surgery, Department of Surgery, Wan Fang Hospital, Taipei Medical University, Taipei, Taiwan; 12 Evidence-based Medicine Center, Wan Fang Hospital, Taipei Medical University, Taipei, Taiwan; Jinling Clinical Medical College of Nanjing Medical University, CHINA

## Abstract

**Background:**

The evidence supporting the benefit of femoral nerve block (FNB) for positioning before spinal anesthesia (SA) in patients suffering from a femur fracture remains inconclusive. In the present study, the authors intended to determine the efficacy and safety of FNB versus an intravenous analgesic (IVA) for positioning before SA in patients with a femur fracture.

**Method:**

PubMed, EMBASE, Cochrane, and Scopus databases were searched up to January 2018. We included randomized controlled studies (RCTs) and observational studies that compared FNB versus IVA for the positioning of patients with femur fracture receiving SA. The primary outcome was pain scores during positioning within 30 min before SA. Secondary outcomes were the time for SA, additional analgesic requirements, anesthesiologist’s satisfaction with the quality of positioning for SA, participant acceptance, and hemodynamic changes. A random-effects model was used to synthesize the data. We registered the study at PROSPERO with an ID of CRD42018091450.

**Results:**

Ten studies with 584 patients were eligible for inclusion. FNB achieved significantly lower pain scores than IVA during positioning within 30 min before SA (pooled standardized mean deviation (SMD): -1.27, 95% confidence interval (CI): -1.84 to -0.70, *p* < 0.05). A subgroup analysis showed that the analgesic effect was larger in patients in the sitting position for SA than a non-sitting position (sitting position vs non-sitting: pooled SMD: -1.75 (*p* < 0.05) vs -0.61 (not significant). A multivariate regression showed that the analgesic effect was also associated with age and the total equivalent amount as lidocaine after adjusting for gender (age: coefficient 0.048, *p* < 0.05; total equivalent amount as lidocaine: coefficient 0.005, *p* < 0.05). Patients receiving FNB also had a significantly shorter time for SA, greater anesthesiologist satisfaction, and higher patient acceptance than patients receiving IVA. The use of local anesthetics did not produce significant clinical hemodynamic change.

**Conclusion:**

Compared to IVA, FNB was an effective and safe strategy for the positioning of femur fracture patients for a spinal block, particularly patients who received SA in the sitting position.

## Introduction

Femoral fracture is a well-known reason for surgical repair in patients of all ages. Sex- and age-standardized incidences of femoral fracture for the UK, German, Netherlands, Danish, and Spanish databases vary from 9 to 52 per 10,000 person-years for the general population [1]. The incidence of femoral shaft fractures ranges from of 9.5 to 18.9 per 100,000 annually [2]. Approximately 250,000 proximal femur fractures occur in the United States annually. This number is anticipated to double by the year 2050 [3]. Approximately 98% of femur fractures are managed surgically [4]. Spinal anesthesia (SA) is the preferred and commonly used method for surgery, and it is associated with a lower odds of mortality compared to general anesthesia [5]. The main proposed reason for improved mortality included avoidance of intubation and mechanical ventilation, decreased blood loss, and improved postoperative analgesia [6]. SA must be administered in a lateral decubitus or sitting position. Movement of the overriding fractured end of the femur is inevitable, which causes excessive pain that makes positioning patients with a fractured femur for SA challenging.

Femoral fractures are a considerably painful bone injury because the periosteum exhibits the lowest pain threshold of all deep somatic structures [7]. Approximately one-third of patient with fractured hip have mild pain at rest, one-third have moderate pain and one-third have severe pain. But, over three-quarters of these patients have moderate to severe pain at movement [4]. The failure to effectively control the pain before surgery in femur fracture patients may lead to potential risks of cardiovascular events. Non-steroidal anti-inflammatory drugs and opioids are commonly used analgesics. However, these agents may cause undesirable side effects and complications [8]. Therefore, proper management of pain with the other choice is paramount.

Femoral nerve block (FNB) is a safe, simple and easy to learn. Local anesthetic is injected through the landmark method or under ultrasound guidance. A recent systematic review of eight trials with 373 participants showed that peripheral nerve block reduces pain on movement within 30 min of block placement more effectively than an intravenous analgesic (IVA) [9]. But heterogeneity is high, and most of these trials used a fascia iliaca nerve block (FINB) and do not specifically evaluate positioning for SA.

Increasing numbers of published studies compared FNB to IVA with femur fractures for positioning for SA [10–19]. But this evidence is not well-integrated. Therefore, we performed a meta-analysis to specifically assess the efficacy and safety of FNB versus IVA for positioning f SA in patients with a femur fracture in the operative setting.

## Methods

Our meta-analysis followed the preferred reporting items for systematic review and meta-analyses (PRISMA) guidelines (S1 PRISMA checklist) [20]. We registered the analysis at PROSPERO (PROSPERO ID: CRD42018091450).

### Search strategy

Two authors (CWH and YPH) independently searched PubMed, EMBASE, Cochrane library and Scopus databases from the first record to January 2018 using eligibility criteria with the following search terms: femoral block, analgesic, spinal anesthesia, and fracture (S1 Table). No language restrictions were applied. We also identified other studies using the reference sections of relevant papers and correspondence with subject experts. We used the ClinicalTrials.gov registry (http://clinicaltrials.gov/) for unpublished studies.

### Inclusion and exclusion criteria

We included all published human randomized control trials (RCTs) or observational studies with an adequate control group. Participants with a femur fracture who received FNB compared to IVA for positioning before a spinal block in an operative setting were included. Case reports, case series, and abstracts were excluded.

### Outcomes of interest

Our primary outcome of interest was pain scores during positioning before SA. Secondary outcomes were the time for SA, additional analgesic requirements, anesthesiologist’s satisfaction with the quality of positioning for SA, participant acceptance, and hemodynamic changes.

### Data extraction and management

Two reviewers (CWH and YPH) independently performed data extraction. Baseline and outcome data, including study design, characteristics of study population criteria for inclusion and exclusion, intervention method, post-treatment parameters, and complications, were extracted. A third reviewer (CC) resolved any discrepancies. We contacted the authors of the studies for additional information if required.

### Assessment of risk of bias

Two reviewers (CHB and WCH) independently assessed the methodological quality. We used the Cochrane risk of bias tool for RCTs [21]. This tool includes six domains: adequacy of randomization, allocation concealment, blinding of participants and personnel, blinding of outcome assessors, incomplete outcome data, as well as reporting bias and other biases. We used the Newcastle-Ottawa scale tool for observational studies [22]. This tool has three domains, including selection of the cohort, comparability of the groups, and quality of the outcomes. The results were summarized in a risk of bias table. We resolved any disagreements on the methodological quality assessment through comprehensive discussions.

### Statistical analysis

Data were analyzed using Review Manager (version 5.3, Copenhagen, Denmark). Pairwise meta-analyses were performed for each included outcome using a random-effects model. We used the mean differences (MD) and 95% confidence interval (CI) to estimate continuous outcomes. The standardized mean difference (SMD) and 95% CI was used when continuous data were given on different scales. For the SMD, we considered 0.2 a small effect, 0.5 a medium effect, and 0.8 a large effect [23]. For binary outcomes, we estimated the odds ratio (OR) with the 95% CI. Significant differences between groups were set at two-sided *p*-values smaller than 0.05. Statistical heterogeneity was estimated using the I² statistic and χ2 test. Based on I² values, statistical heterogeneity was categorized into low (< 30%), moderate (30% - 60%), or high (> 60%) [24]. If substantial heterogeneity was identified, we explored potential causes using prespecified subgroup analyses (study design, country, fracture type, American Society of Anesthesiologists (ASA) classification, local anesthetic, SA position, and time from intervention to SA). We also performed a sensitivity analysis to better understand the sources of statistical heterogeneity between studies and tested the robustness of our findings based on the RCTs that were excluded because of high or unclear risk in each domain of the risk of bias, RCTs that were excluded because of unclear information on the time from trauma to surgery or body weight, and RCTs that were excluded because of no use of a stimulator to assist FNB or ultrasound to guide FNB. Outcome measures were cross-validated using the mean difference. We applied a meta-regression to assess relationships between age, gender, the total equivalent concentration as lidocaine (calculated as follows: lidocaine = 1, bupivacaine = 4, and ropivacaine = 3) [25], total equivalent amount as lidocaine (calculated as the total equivalent concentration in lidocaine multiplied by the applied volume), and primary outcome using Comprehensive Meta-Analysis software (version 3.3.070, Biostat, Inc., Englewood, New Jersey, USA). To calculate the power for a random-effects meta-analysis, we used anticipated summary effect size (SMD = 0.8, i.e. large effect) based on the study reported by Guay et al. [9] and the finding of our previous work [26]. The average number of participants per group, total number of effect sizes, and study heterogeneity were calculated based on current meta-analysis results. The power analysis was performed according to the method reported by Valentine et al. [27]. Given a two-sided type I error of 0.05, power large than 90% (i.e.βerror less than 10%) was regarded as powerful. If at least 10 studies were included, asymmetry in funnel plots was used to detect publication bias. We estimated the possible small study effects using Egger’s test [28].

## Results

### Results of the search

**Fig 1:**
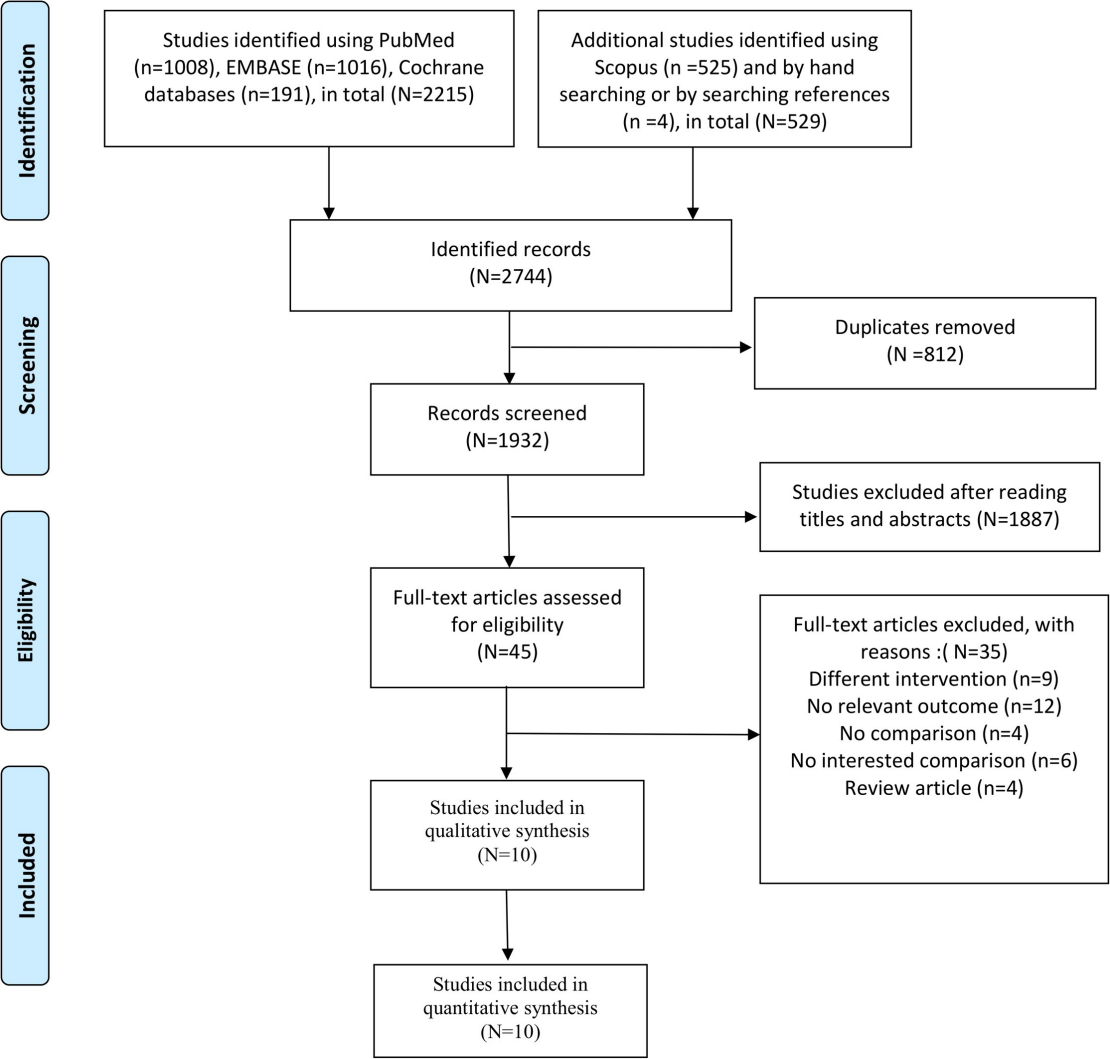
Flow diagram of the search process and search results.

Fig 1 illustrates the screening and selection processes for the included studies. We identified 2,744 potentially relevant records through multiple database searches (PubMed, EMBASE, and Cochrane library (n = 2,215); Scopus) and by searching references (n = 529). A total of 1,932 studies remained after removing duplicate articles. We screened the titles and abstracts, and 1,887 articles were determined to be ineligible. Full-text articles were excluded with no comparison (*n* = 4), no comparison of interest (*n* = 6), different interventions (*n* = 9), no relevant outcome measure (*n* = 12), and review articles (*n* = 4). Ten studies [10–19] were ultimately included for qualitative and quantitative synthesis.

### Study characteristics

Tables 1 and S2 show the characteristics of the included studies. Eight studies [10–12, 14–18] were RCTs, and two studies [13, 19] observational studies. These studies were performed in single centers in Italy [16], Ireland [18], Pakistan [10], India [12, 13, 15, 17, 19], Nepal [14], and Thailand [11]. The study sample sizes ranged 24–100 subjects with 584 subjects in total. The average age of participants ranged 32–80.2 years. Three of the included studies [14, 16, 18] recruited more males than females for FNB. Two [10, 12] of the studies recruited a majority of females for FNB. Four [11, 15, 17, 19] studies recruited equal numbers of the two sexes, and one [13] study provided no information on sex. Three trials [10, 11, 16] reported the time from trauma to surgery, and averages ranged from 2.3–15.6 days. Two studies included an isolated femoral neck fracture [16, 18], one study included only femoral shaft fractures [13], and the other studies included all femur fractures [10–12, 14, 15, 17, 19]. Participants were ASA I, II or I-III in various proportions. Exclusion criteria were similar for those in the included studies. For the method of FNB, time from the intervention to SA varied from 5 to 30 min. Most studies used the landmark method [10–13, 15–19] or a stimulator [11–19] to perform FNB. The doses and types of local anesthetics were different. Seven studies [10, 12, 14–16, 18, 19] used lidocaine, two [11, 13] studies used bupivacaine, and one [17] study used ropivacaine. The dose and type of IVA also varied. Nine studies [11–19] used fentanyl, and one study [10] used nalbuphine. For the position for SA, six studies [10, 13, 14, 16–18] used the sitting position, and four studies [11, 12, 15, 19] did not use the sitting position.

**Table 1:** Characteristics of the included studies.

Study	Country	Design	Sample size		Inclusion criteria			FNB method			Regiment		SA position
			FNB	IVA	ASA	Age (years)	Fracture type	Time from FNB to SA	Approach	Guidance	FNB	IVA	
Sia 2004 [16]	Italy	RCT, 1 center	20	20	I, II	NA	Femoral neck	5 min	Landmark	Stimulator	L, 15 mL 1.5%	F, 3 µg/kg IV	Sitting
Szucs 2012 [18]	Ireland	RCT, 1 center	12	12	I~III	>50	Femoral neck	15 min	Landmark	Stimulator	L, 10 ml 2%	F, NA	Sitting
Durrani 2013 [10]	Pakistan	RCT, 1 center	42	42	I, II	18–80	Femur	15 min	Landmark	No guidance	L, 15 ml, NA% with adrenaline	Nal, 6 mg IV	Sitting
Jadon 2014 [12]	India	RCT, 1 center	30	30	I~III	15–70	Femur	5 min	Landmark	Stimulator	L, 20 mL, 1.5% with adrenaline	F, 1 µg/kg IV	Not sitting
Reddy 2016 [15]	India	RCT, 1 center	36	36	I~III	18–70	Femur	5 min	Landmark	Stimulator	L, 20 mL, 1.5% with adrenaline	F, 1 µg/kg IV	Not sitting
Ranjit 2016 [14]	Nepal	RCT, 1 center	20	20	I, II	18–75	Femur	5 min	Ultrasound	Stimulator	L, 20 mL, 1.5% with adrenaline	F, 2 µg/kg IV	sitting
Vats 2016 [19]	India	Observational study, 1 center	50	50	I-II	18–70	Femur	5 min	Landmark	Stimulator	L, 20 mL, 1.5% with adrenaline	F, 1 µg/kg IV	Not sitting
Iamaroon 2010 [11]	Thailand	RCT, 1 center	32	32	I-II	18–75	Femur	15 min	Landmark	Stimulator	B, 30 ml 0.3%	F, 0.5 µg/kg IV, two doses with a 5-min interval	Not sitting
Pakhare 2016 [13]	India	Observational study, 1 center	30	30	I-II	18–65	Femoral shaft	30 min	Landmark	Stimulator	B, 30 ml 0.3%	F, 3 µg/kg IV	Sitting
Singh 2016 [17]	India	RCT, 1 center	30	30	I-II	18–70	Femur	15 min	Landmark	Stimulator	R, 15 ml 0.2%	F, 0.5 µg/kg IV	Sitting

RCT, randomized controlled trial; FNB, femoral nerve block; IVA, intravenous analgesic; NA, not available; ASA, American Society of Anesthesiologists; SA, spinal anesthesia; L, lidocaine; B, bupivacaine; R, ropivacaine; F, fentanyl; Nal, Nalbuphine; IV, intravenous.

#### Assessment of the risk of bias

Table 2 describes the methodological quality of the identified studies. Most RCT studies were rated as low risk of bias of randomization, incomplete outcome data, and selective reporting. Three studies [12, 15, 16] had unclear information on allocation and concealment. But we rated all studies [10–19] as an unclear or high risk of performance bias because of the intervention method used. We also rated three studies [14–16] as having a high risk of bias because these studies did not perform prespecified sample size calculations. Two of the observational studies [13, 19] were identified. We rated one study [13] as moderate quality (NOS score: 6), and the other study [19] as high quality (NOS score: 8).

**Table 2:** Risk of bias assessment for included studies.

Cochrane risk of bias assessment for randomized controlled trials													
Study	Randomization			Allocation and concealment			Blinding of participant and study personnel	Blinding of outcome assessor		Selective outcome reporting	Reporting bias		Other bias
Sia 2004 [16]	Unclear			Unclear			Unclear	Low		Low	Low		High$
Durrani 2013 [10]	Low*			Low#			Unclear	Unclear		Low	Low		Low
Jadon 2014 [12]	Low			Unclear			Unclear	Unclear		Low	Low		Low
Reddy 2016 [15]	Low*			Unclear			Unclear	Unclear		Low	Low		High$
Ranjit 2016 [14]	Low*			Low#			Unclear	Unclear		Low	Low		High$
Iamaroon 2010 [11]	Low*			Low#			High	Low		Low	Low		Low
Szucs 2012 [18]	Low*			Low#			Unclear	Unclear		low	low		Low
Singh 2016 [17]	Low*			Low#			Unclear	Unclear		low	low		Low
Newcastle-Ottawa scale& (NOS) for assessment of observational studies													
Study, year	Selection							Comparability		Outcome			Total score
	Representativeness of the exposed cohort		Selection of the non-exposed cohort		Ascertainment of exposure		Demonstration that outcome of interest was not present at start of study	Comparability of cohorts on the basis of the design or analysis		Assessment of outcomes	Was follow-up long enough for outcomes to occur?	Adequacy of follow-up of cohorts	
								Age	Sex				
Vats 2016 [19]	Truly ★		Same community ★		Good ★		Yes ★	Yes ★	Yes ★	No description -	Yes ★	Complete follow-up ★	8
Pakhake 2016 [13]	Truly ★		Same community ★		Good ★		Yes ★	No -	No -	No description -	Yes ★	Complete follow-up ★	6

* Random number table;

^#^ sealed envelope;

^$^ No prespecified sample size calculation;

^★^one star indicates 1 score;

^&^ The NOS is a nine-point scale with a maximum of four points allocated to selection, two points for comparability, and three points for outcome. Studies scoring ≥7 were considered high quality; 4~6, moderate quality; and ≤4, low quality.

### Primary outcomes

#### Pain scores during positioning before SA (within 30 min)

**Fig 2:**
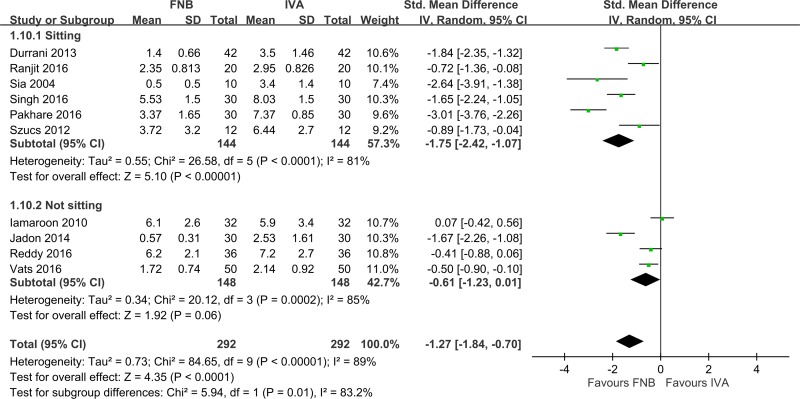
Forest plot of positioning before spinal anesthesia (within 30 min).

**Fig 3:**
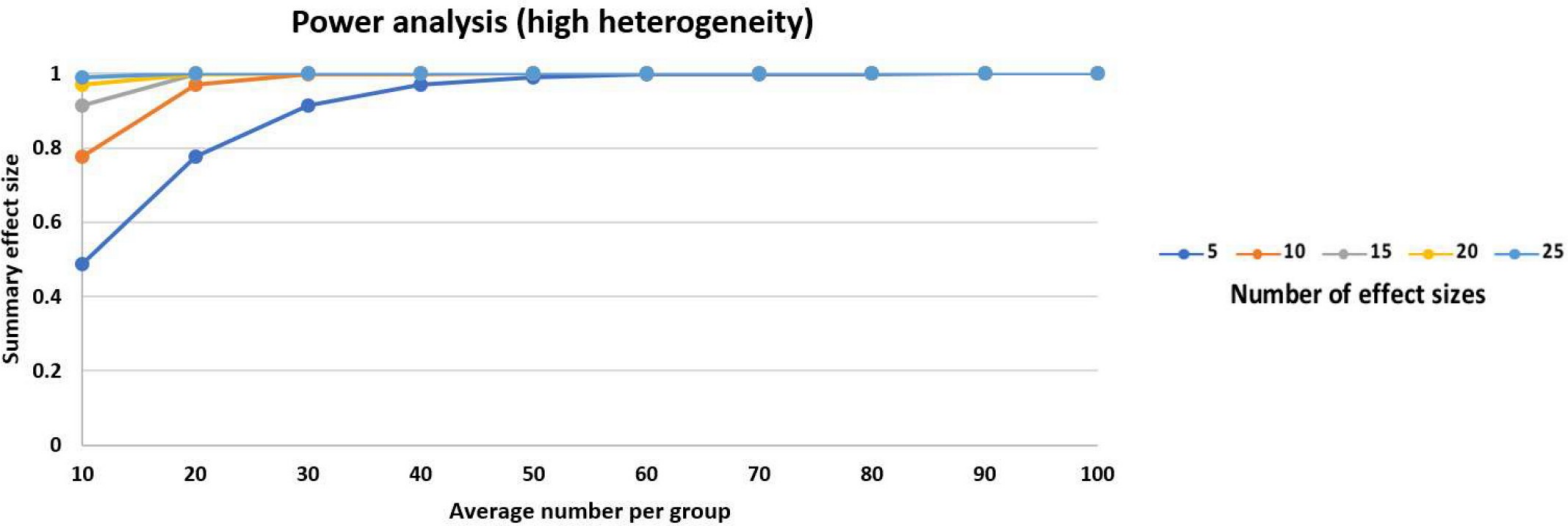
Power analysis for meta-analysis. The power was calculated as 0.9963 based on the anticipated effect size of SMD = 0.8, the 10 identified studies, average of 29 participants per group and high heterogeneity between studies.

All ten studies [10–19] (*n* = 584) evaluated pain scores during positioning within 30 min before SA (Fig 2). FNB achieved significantly lower pain scores than IVA (pooled SMD: -1.27, 95% CI: -1.84 to -0.70, *p* < 0.05, I^2^ = 89%). This finding was powerful (Fig 3).

**Fig 4:**
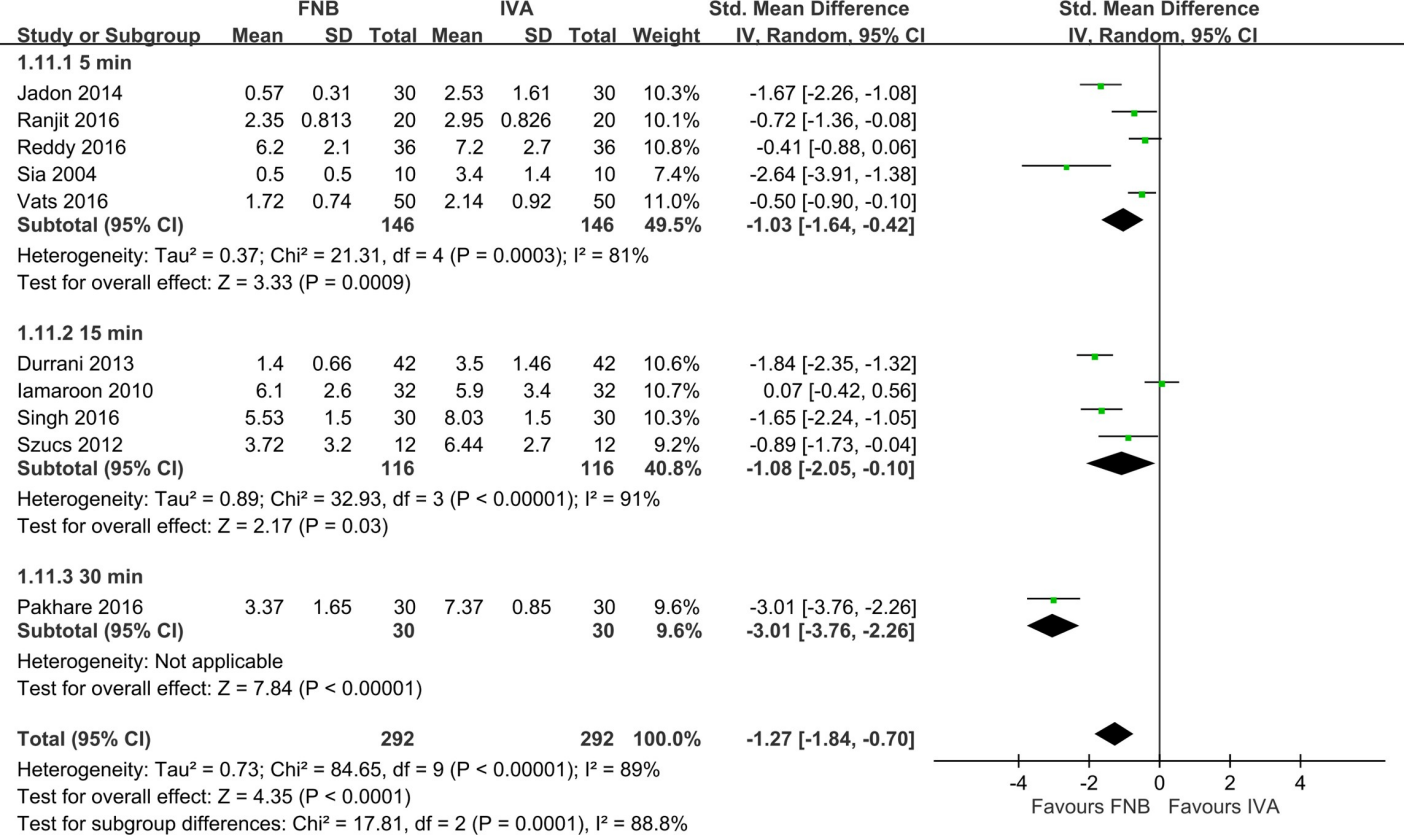
Forest plot for comparisons of pain scores during positioning before spinal anesthesia (within 30 min) subgroup by time from intervention to spinal anesthesia.

A subgroup analysis showed that pain scores reduction changed with the SA position and time from FNB to SA. Pain scores reduction was not influenced by the study design, country, fracture type, ASA classification, or type of local anesthetic (Table 3). FNB had larger pain score reductions than IVA in the sitting position (Fig 2, six studies (*n* = 288), pooled SMD: -1.75, 95% CI: -2.42 to -1.07, *p <* 0.05, I^2^ = 81%). However, FNB had smaller pain score reductions than IVA in a non-sitting position, but this effect was statistically insignificant (Fig 2, four studies (*n* = 296), pooled SMD: -0.61, 95% CI: -1.23 to 0.01, *p* = 0.06, I^2^ = 85%). Notably, heterogeneity remained high. As to the time from FNB to SA, FNB had larger pain score reductions in 30 min than 5 or 15 min (Fig 4, pooled SMD: 30 min (-3.01, *p <* 0.05) vs. 5 min (-1.03, *p <* 0.05) and 15 min (-1.08, *p <* 0.05)). However, the results for 30 min were collected from only one study [17], which makes this estimate less reliable.

**Table 3:** Predefined clinical subgroup analysis with pain scores during positioning comparing femoral nerve block with intravenous analgesic.

Category	Subgroups	No. of studies	No. of patients	SMD (95% CI)	*p* value	Group heterogeneity		Subgroup difference	
						I^2^	*p* value	I^2^	*p* value
Outcome: pain scores during positioning									
Study design	RCT	8	424	-1.15 (-1.74, -0.57)	<0.05	86	<0.0001	0	0.65
	Observational study	2	160	-1.73 (-4.19,0.73)	0.17	97	<0.0001		
Country	India	5	352	-1.41 (-2.25, -0.57)	<0.05	92	<0.0001	0	0.65
	Not India	5	232	-1.13 (-2.02.-0.24)	<0.05	89	<0.0001		
Fracture type	Isolated Femoral neck Fracture	2	44	-1.70 (-3.41,0.02)	0.05	80	< 0.05	0	0.58
	Femur fracture	8	584	-1.19 (-1.82, -0.56)	<0.05	91	<0.0001		
ASA classification	I-II	6	428	-1.79 (-2.86, -0.73)	<0.05	96	<0.0001	0	0.93
	I-III	4	156	-1.74 (-2.74, -1.00)	<0.05	29	0.24		
Local anesthetic	Lidocaine	7	400	-1.15 (-1.69, -0.61)	<0.05	83	<0.0001	0	0.48
	Ropivacaine	1	60	-1.65 (-2.24, -1.05)	<0.05	NA	NA		
	Bupivacaine	2	124	-1.46 (-4.45,1.55)	0.34	98	<0.0001		
SA position	Sitting	6	288	-1.75 (-2.42, -1.07)	<0.05	81	<0.0001	83.2	0.01^*^
	Not sitting	4	296	-0.61 (-1.23,0.01)	0.06	85	<0.0001		
Time from intervention to SA	5 min	5	292	-1.03 (-1.64, -0.42)	<0.05	81	0.0003	88.8	< 0.05^*^
	15 mins	4	232	-1.08 (-2.05, -0.10)	<0.05	91	<0.0001		
	30 min	1	60	-3.01 (-3.76, -2.26)	<0.05	NA	NA		

ASA, American Society of Anesthesiologists; CI, confidence interval; RCT, randomized control trial; SA, spinal anesthesia; SMD, standardized mean difference;

*, statistically significant.

**Fig 5:**
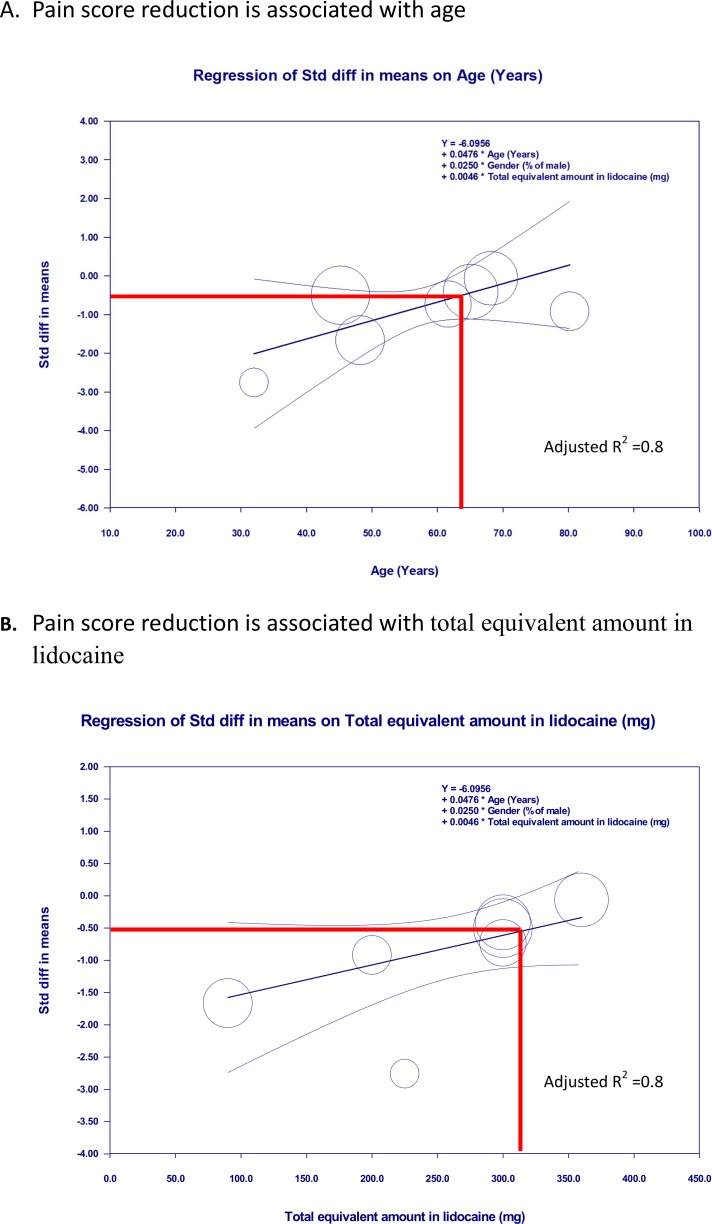
Meta-regression for standardized mean difference (SMD) of pain scores during positioning for spinal anesthesia between femoral nerve block and intravenous analgesics. A. The SMD is proportional to the age of the patient. B. The SMD is proportional to the total equivalent amount as lidocaine, i.e., FNB using a low total equivalent amount as lidocaine or use in younger patients was associated with more analgesic effect than IVA after adjusting for gender.

We performed a meta-regression to determine whether the effect size varied with age, gender, total equivalent concentration as lidocaine, and total equivalent amount as lidocaine. The SMD for FNB compared to IVA was not moderated by age (*p* = 0.21), gender (*p* = 0.23), total equivalent concentration as lidocaine (*p* = 0.52), or total equivalent amount as lidocaine (*p* = 0.58) according to a univariate regression model (Table 4). After adjusting for gender, age, or the total equivalent amount as lidocaine, both factors showed a positive association with SMD for pain scores (Table 5, models 1, 2, and 5), but the total equivalent concentration as lidocaine showed no association with the primary outcome after adjusting for gender (Table 5, models 2 and 3). Model 5 was the best model for predicting the association with effect sizes after adjusting for gender (Table 5, model 5, adjusted *R*^2^ = 0.80). The SMD of pain scores were increased by 0.048 for every year increase in patient age (*p *< 0.05). The SMD of pain scores were increased by 0.005 for every milligram increase in the total equivalent amount as lidocaine (*p* < 0.05) (Fig 5).

**Table 4:** Univariate meta-regression predicting estimates of pain scores during positioning between femoral nerve block and intravenous analgesic.

Covariate	No. of study	Univariate analysis		
		Coefficients (95% CI)	p-value	Adjusted R^2^
Gender (% of male)	9	-0.018 (-0.048–0.011)	0.23	0.06
Age (years)	9	0.030 (-0.017–0.076)	0.21	0
Equivalent amount in lidocaine	9	0.059 (-0.122–0.239)	0.52	0
Equivalent concentration in lidocaine	9	0.002 (-0.005–0.010)	0.58	0

CI, confidence interval.

**Table 5:** Multivariate meta-regression models predicting estimates of pain scores during positioning.

Covariate	Multivariate analysis														
	Model 1 (No. of study = 8)			Model 2 (No. of study = 8)			Model 3 (No. of study = 8)			Model 4 (No. of study = 7)			Model 5 (No. of study = 7)		
	Coefficient (95% CI)	p	Adjusted R^2^	Coefficient (95% CI)	p	Adjusted R^2^	Coefficient (95% CI)	p	Adjusted R^2^	Coefficient (95% CI)	p	Adjusted R^2^	Coefficient (95% CI)	p	Adjusted R^2^
Gender (% of male)	0.035 (0.015–0.085)	0.17	0.28	-0.014 (-0.039–0.011)	0.28	0.38	-0.014 (-0.050–0.022)	0.44	0	0.04 (-0.018–0.098)	0.18	0	0.024 (-0.011–0.060)	0.18	0.80
Age(years)	0.077 (0.014–0.141)	0.02^*^		NA	NA	NA	NA	NA	NA	0.076 (0.006–0.146)	0.03^*^		0.048 (0.001–0.096)	0.04^*^	
Equivalent amount in lidocaine	NA	NA	NA	0.006 (0.001–0.011)	0.03^*^	0.38	NA	NA	NA	NA	NA	NA	0.005 (0.001–0.009)	0.02^*^	
Equivalent concentration in lidocaine	NA	NA	NA	NA	NA	NA	0.003 (-0.168–0.173)	0.97	0	0.008 (-0.160–0.175)	0.93	0	NA	NA	NA

_NA, no analysis;_

_*, statistically significant._

**Fig 6:**
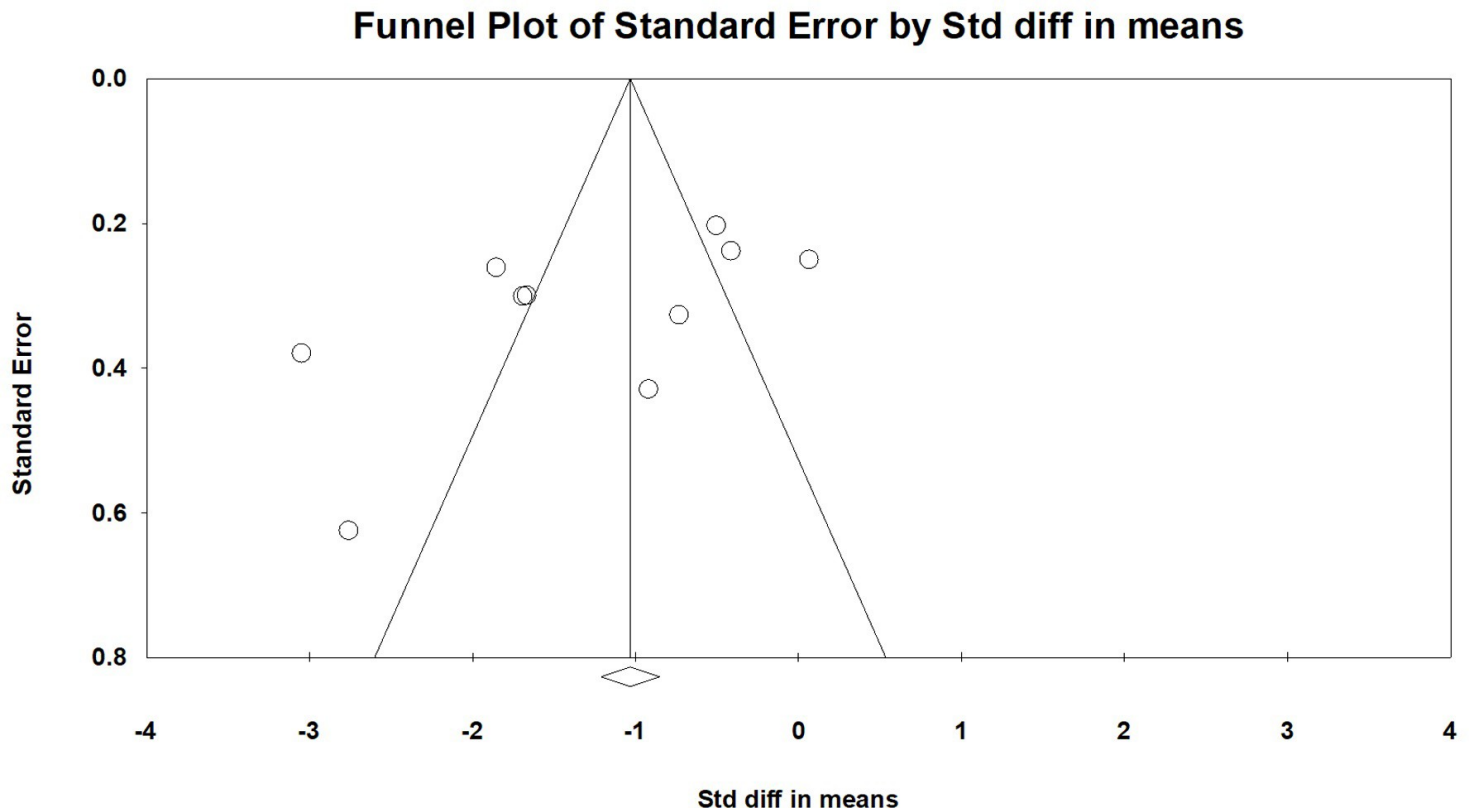
Funnel plots for the comparisons of FNB with IVA on pain scores during positioning. Standardized mean difference against standard error for 10 simulated studies of varying sample size where there is no publication bias.

We performed a sensitivity analysis to assess the robustness of our findings based on RCTs that were excluded because of high or unclear risk in each domain of the risk of bias, RCTs that were excluded because of unclear information about the time from trauma to surgery (days) or body weight, RCTs that were excluded because of no use of a stimulator, and RCTs that were excluded because of no use of ultrasound guidance. These factors did not influence our findings (Table 6). We used the mean difference to cross-validate the outcome measure. This result also indicated that our findings were robust. The funnel plots that showed no asymmetry, which indicates no evidence of a small study effect (Fig 6, Egger’s test: not significant).

**Table 6:** Sensitivity analyses: The effect of potential biases on primary outcomes.

Potential bias or limitations excluded	No of studies	No of patients	SMD (95% CI)	I^2^ (%)	P value	MD (95% CI)	I^2^ (%)	*p* value
Overall	10	584	-1.27 (-1.84, -0.70)	89	<0.0001	-1.79 (-2.62, -0.96)	94	< 0.0001
RCT quality^a^								
RCT overall	8	424	-1.15 (-1.74, -0.57)	86	0.0001	-1.70 (-2.38, -1.01)	83	< 0.0001
Randomization	7	404	-1.01 (-1.60, -0.42)	87	0.0008	-1.52 (-2.22, -0.81	82	< 0.0001
Allocation and concealment	5	272	-0.98 (-1.24, -0.72)	88	<0.00001	-1.52 (-1.83, -1.21)	87	< 0.0001
Blinding of outcome assessor	2	84	-1.22 (-3.87,1.43)	93	0.37	-1.44 (-4.44,1.63)	92	0.36
Other bias	5	292	-1.20 (-1.99, -0.40)	89	0.003	-1.92 (-2.54, -1.30)	62	< 0.0001
Participants^b^								
Time from trauma to surgery (days)	3	168	-1.40 (-2.98,0.17)	94	0.08	-1.76 (-3.04, -0.47)	84	< 0.01
Weight(kg)	4	216	-1.05 (-2.01, -0.08)	90	0.03	-1.54 (-2.61, -0.48)	80	< 0.01
Method								
FNB with stimulator^c^	9	500	-1.20 (-1.81, -0.59)	89	0.0001	-1.75 (-2.71, -0.81)	94	< 0.0001
FNB by landmark^d^	9	544	-1.33 (-1.96, -0.70)	90	< 0.001	-1.94 (-2.87.-1.01)	94	< 0.0001

a, excluded high or unclear risk;

b, excluded with unclear information for time from trauma to surgery (days), body weight (kg),

c, excluded with no use of stimulator;

d, excluded use of ultrasound guidance;

CI, confidence interval; SMD, standardized mean difference; MD, mean difference

#### Secondary outcomes

**Fig 7:**
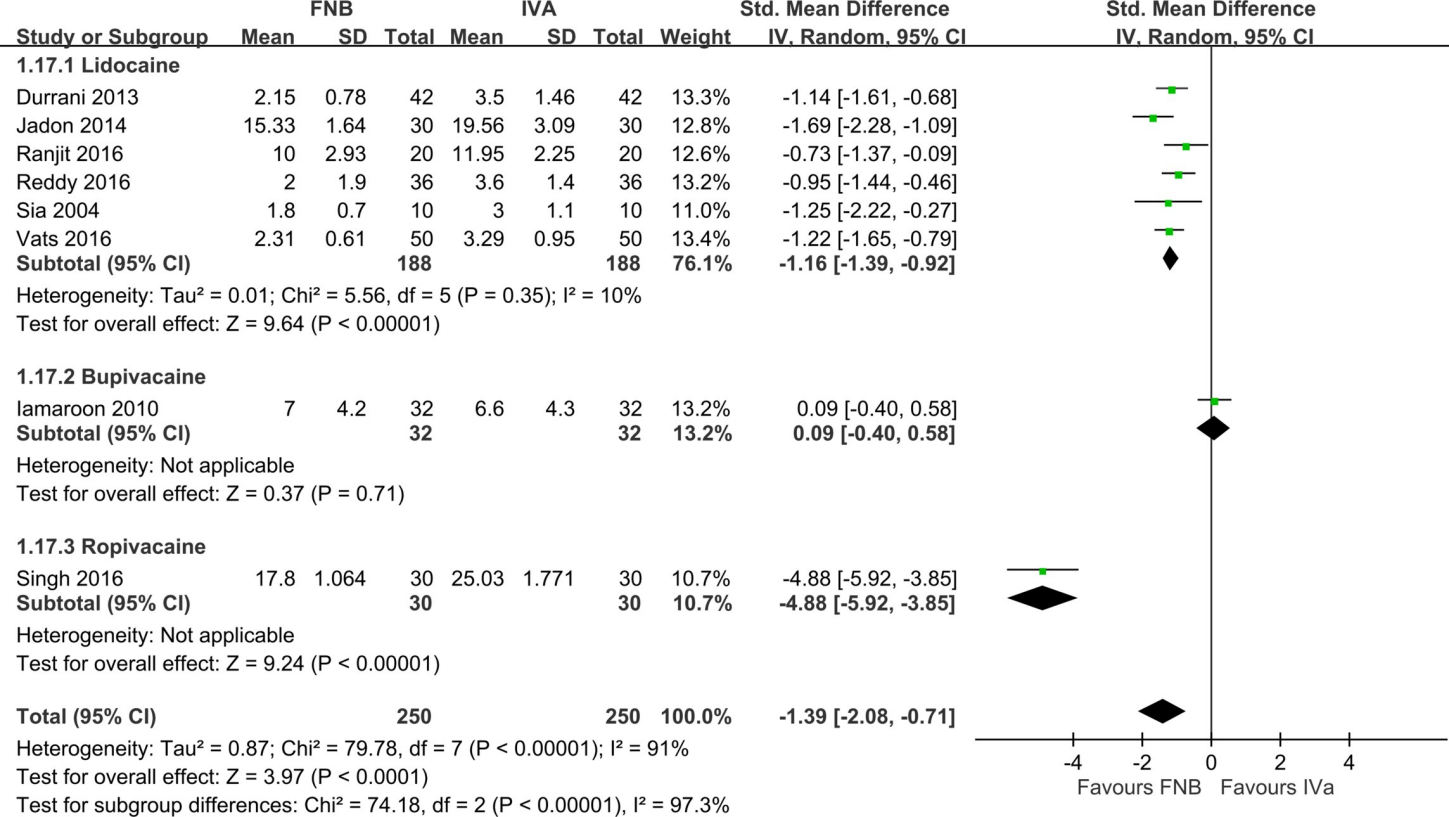
Forest plot of time for spinal anesthesia (min). FNB, femoral nerve block; IVA, intravenous analgesic.

**Fig 8:**
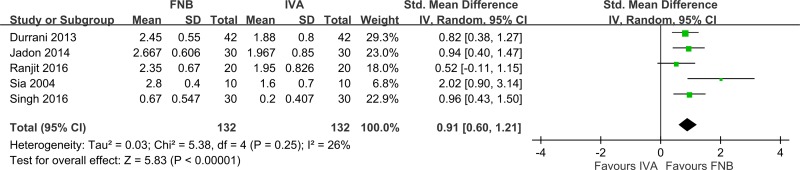
Forest plot of anesthesiologist’s satisfaction with the quality of spinal anesthesia. FNB, femoral nerve block; IVA, intravenous analgesic.

**Fig 9:**
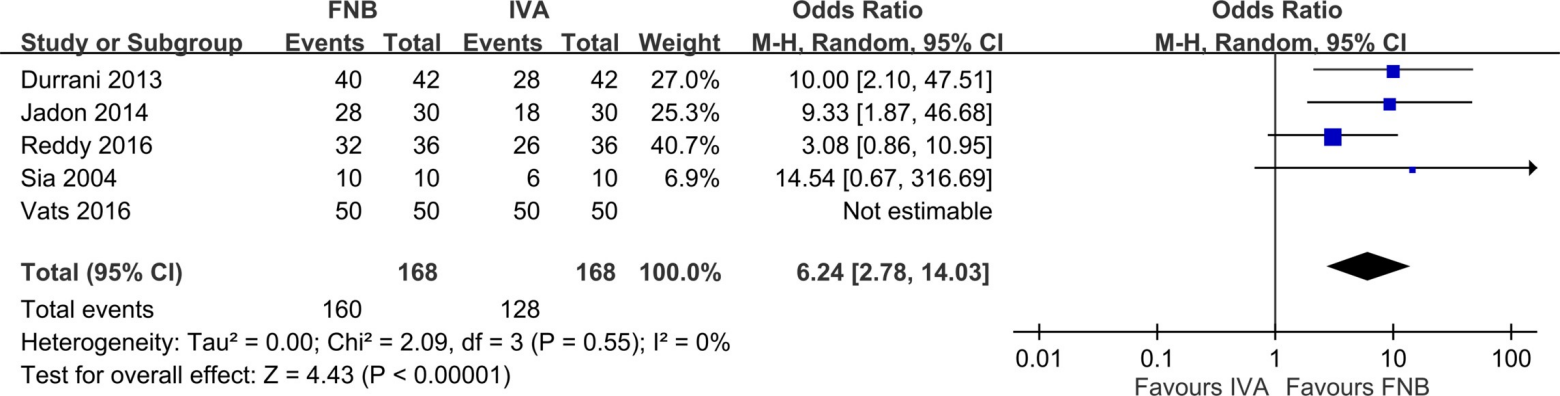
Forest plot of participant acceptance. FNB, femoral nerve block; IVA, intravenous analgesic.

**Fig 10:**
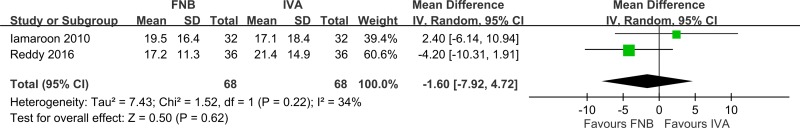
Forest plot of additional analgesic requirements. FNB, femoral nerve block; IVA, intravenous analgesic; CI, confidence interval.

Compared to IVA, patients who received FNB had a significantly shorter time for SA (Fig 7, eight studies [10–12, 14–17, 19], 500 patients; pooled SMD: -1.39; 95% CI: -2.08 to -2.71, *p <*0.05; I^2^ = 91%). Heterogeneity was high, which resulted from the type of local anesthetic (Singh 2016 [17] (ropivacaine); Iamaroon 2010 [11] (bupivacaine); and others [10, 12, 14–16, 19] (lidocaine)). Anesthesiologists were more satisfied with FNB than IVA (Fig 8, five studies [10, 12, 14, 16, 17], 264 patients; pooled SMD: 0.91; 95% CI: 0.60 to 1.21, *p <* 0.05; I^2^ = 26%). Participants preferred FNB for analgesia (Fig 9, five studies [10, 12, 15, 16, 19], 336 patients; pooled OR: 6.24; 95% CI: 2.78 to 14.03, *p <* 0.05; I^2^ = 0%). Patients who used FNB also had lower additional analgesic requirements than patients who used IVA, but the effect was small and not statistically significant (Fig 10, two studies [11, 15] 136 patients; pooled SMD: -0.10; 95% CI: -0.54 to 0.34, *p = 0*.*62*; I^2^ = 41%).

### Safety outcomes

Compared to IVA, FNB using lidocaine had a slightly higher mean MAP by 3.11 mmHg (S1 Fig, four studies [12, 14, 15, 19], 272 patients; pooled MD: 3.11; 95% CI: 0.18 to 6.05, *p <* 0.05; I^2^ = 61%). Pakhare et al. [13] used bupivacaine for FNB and reported a lower mean MAP by 4.30 mmHg (*n* = 60, 95% CI: -6.16 to -2.44, *p <0*.*05*). No significant difference in mean heart rate was detected between FNB using lidocaine and IVA (S2 Fig, four studies [12, 14, 15, 19], 272 patients; pooled MD: 0.99 beats/min; 95% CI: -1.31 to 3.11, *p =* 0.99; I^2^ = 0%). Pakhare et al. [13] used bupivacaine for FNB and reported a lower mean heart by 11.61 beats/min (*n* = 60, 95% CI: -14.49 to -8.77, *p <* 0.05). Three studies [12, 14, 15] reported that FNB using lidocaine and IVA provided adequate mean SpO_2_ values (> 94%), as shown in S3 Fig. The safety outcome of interest for FNB using ropivacaine was lacking.

## Discussion

The main findings of the present meta-analysis were that 10 studies [10–19] comprising 584 participants showed that FNB was superior to IVA. FNB resulted in significantly lower pain scores during positioning within 30 min before SA. Eight studies [10–12, 14–17, 19] (500 participants) showed that FNB also reduced the time to perform SA compared to IVA. This finding means that FNB provided more effective analgesia, which improved patient positioning. Anesthesiologists and participants preferred FNB for analgesia. Different local anesthetics used for FNB differentially impacted hemodynamic parameters, but the effect was small.

Regarding pain scores during positioning, a previous meta-analysis performed by Guay et al. [9] reported that peripheral nerve block reduced pain on movement within 30 min. But, the heterogeneity in this study was high, and the effect was associated with the total equivalent concentration as lidocaine. This result was limited by the including of patients from various clinical situations with different types of nerve block. Our meta-analysis focused on positioning patients with a femur fracture for a spinal block in the operative setting. We found that the result of the analgesic effect was consistent with Guay et al. [9]. However, an association between the analgesic effect and total equivalent concentration as lidocaine was not observed in the univariate or multivariate regression model in our study. Our study found that the analgesic effect was associated with age, the total equivalent amount as lidocaine or both, after adjusting for gender. The reasons for the discrepancy with the previous study are that these studies included patients from different clinical entities, inadequate analgesia of IVA secondary by dose differences and variation of IVA, the time to evaluated the pain scores, the use of different volumes of local anesthetics and different baseline characteristics. We found that FNB achieved a larger than median analgesic effect that was associated with the use of a total equivalent amount of lidocaine of < 300 mg and patients aged < 63 years (Fig 5). However, this finding should be interpreted with caution because these analyses investigated differences between studies. Whether the application of FNB using a low amount of lidocaine or in patients younger than 63 years produces larger than median analgesic effects is not known. Further well-designed studies are warranted to establish a causal relation.

Another strength of our meta-analysis is that we performed a sensitivity analysis to better recognize the sources of heterogeneity among the included studies. The results indicated that our findings were robust based on RCTs that were excluded because of high or unclear risk in each domain of risk of bias, RCTs that were excluded because of unclear information about the time from trauma to surgery or body weight, and RCTs that were excluded that did not use a stimulator to assist FNB or use ultrasound to guide FNB. Forouzan et al. [29] also reported that ultrasonography- and nerve stimulator-guided FNB exhibited the same success rates and block durations. As to participant factors, only a few studies [10, 11, 16] clearly reported the time from trauma to surgery and body weight. Therefore, we suggest that further studies consider these factors, which may differentially influence the primary outcome.

SA may be performed in a lateral decubitus or sitting position. The decision of which position to use to perform SA is an individual choice that exhibits no obviously clear or consistent regional or even institutional associations. For the impact of positioning patients for SA, a meta-analysis by Zorrilla-Vaca et al. [30] indicated that the lateral decubitus position was associated with a reduction of 39% relative risk on the incidence of post-dural puncture headaches compared to the sitting position. The results of our meta-analysis showed that FNB produced a greater pain score reduction than IVA in the sitting and non-sitting positions, but the effect size was larger in the sitting position group. This difference means that most patients experienced less pain using FNB for patients with femur fractures in the sitting position to approach SA. One explanation is that patients who change from a supine position to sitting may need to flex the hip joint or put traction on the femur fracture, which may elicit more pain than patients changing from a supine position to a non-sitting position, such as with the lateral decubitus position [15, 31].

Concern about the use of nerve blocks to aid positioning may delay operating lists. and the analgesic effect of lidocaine is felt within 5 min, but 20–30 min are required for bupivacaine to exert its effect [32]. Our study found that lidocaine, bupivacaine, and ropivacaine all had larger analgesic effects than IVA. Six [10, 12, 14–16, 19] of the included studies using lidocaine showed homogeneous results in reduced times for SA. Peripheral nerve block may be administered before surgery in the anesthesia induction room with adequate equipment [33]. As mentioned above, we suggest that FNB using lidocaine in the anesthesia induction room may balance this concern.

SA was a preferred and commonly used method for femur fracture surgery. Use of SA was associated with a 25%– 29% reduction in major pulmonary complications and death [5] due to the avoidance of intubation and mechanical ventilation, decreased blood loss, and improved postoperative analgesia [6]. Opioids are commonly used and offer good analgesia in femur fracture patients. However, opioids are notorious for adverse effects, including nausea, vomiting, and respiratory depression [8]. Opioids may also induce delirium in these patients [34, 35]. Diminishing opioid consumption and unnecessary complications are crucial [36]. Our meta-analysis showed that patients using FNB had lower additional postoperative analgesic requirements than patients using IVA, but this effect was small and not statistically significant. This finding was related to only two studies [11, 15] that reported this outcome and included relatively small sample sizes. Guay et al. [9] included seven studies and 285 participants and reported that peripheral nerve block, administered as a single shot or continuous block, resulted in less postoperative opioid requirements compared to no nerve block. Diakomi et al. [37] compared FINB to IVA to facilitate positioning for femur fracture patients under spinal block. Their study results also showed that FINB had lower opioid requirements than IVA. Another meta-analysis by Wang et al. [38] included four studies and showed that FNB and FINB resulted in similar opioid requirements until 48 h postoperatively in patients undergoing total knee and hip arthroplasties. Taken together, we suggest the need for more studies to elucidate the impact of FNB on postoperative opioid requirements.

### Study limitations

Our meta-analysis has some limitations. Because the inclusion of observational studies could bias estimates of the true intervention effect, we performed subgroup analyses and found that the analgesic effect was not significantly different between RCTs and observational studies. Given that pediatric patients were not involved in the identified trials, generalization to that group should be done with great caution. Our meta-analysis was based on ten recently published studies [10–19]. But the sample sizes were relatively small, and these studies were primarily performed in a single center in developing countries. We had insufficient power to demonstrate the effect on additional analgesic requirement. Functional outcomes were not analyzed because of insufficient data. The follow-up periods were too short to analyze long-term complications.

### Conclusion

FNB is an effective strategy that provides significantly better analgesia to facilitate the positioning of femur fracture patients for a spinal block, particularly patients who receive SA in the sitting position. In this meta-analysis, the analgesic effect was associated with the amount of local anesthetics and patient age. FNB also required less time for SA, had lower postoperative opioid requirements, had higher anesthesiologist satisfaction and patient acceptance, and produced no major hemodynamic instabilities.

## Supporting information

S1 PRISMA checklist PRISMA checklist. (DOC)

S1 Table Search strategy. (DOCX)

S2 Table Baseline characteristics of the included studies. (DOCX)

S1 Fig Forest plot of mean arterial pressure. (TIF)

S2 Fig Forest plot of mean heart rate. (TIF)

S3 Fig Forest plot of mean SpO2. (TIF)
