# Supplementary material for: Efficacy and safety of femoral nerve block for the positioning of femur fracture patients before a spinal block – A systematic review and meta-analysis
Source: PLoS One. 2019 May 2;14(5):e0216337. doi: 10.1371/journal.pone.0216337 (PMC6497313; doi:10.1371/journal.pone.0216337)
Supplement: S1 Table — (DOCX) [file pone.0216337.s002.docx]

S1Table. Search Strategy

| Recent queries in PubMed on January 11, 2018 | | |
| --- | --- | --- |
| Search | Query | Items found |
| 1 | Search femoral nerve block | 1756 |
| 2 | Search Fentanyl | 22382 |
| 3 | Search morphine | 54256 |
| 4 | Search tramodol | 3 |
| 5 | Search tramadol | 4491 |
| 6 | Search Nalbuphine | 915 |
| 7 | Search analgesic | 558247 |
| 8 | Search analgesia | 76409 |
| 9 | Search pain | 715708 |
| 10 | Search position | 357313 |
| 11 | Search spinal anesthesia | 23361 |
| 12 | Search spinal block | 27915 |
| 13 | Search regional anesthesia | 69945 |
| 14 | Search subarachnoid block | 23183 |
| 15 | Search fracture | 250105 |
| 16 | Search ((((((((femoral nerve block) OR Fentanyl) OR morphine) OR tramadol) OR Nalbuphine) OR analgesic) OR analgesia) OR pain) OR position | 1544971 |
| 17 | Search (((spinal anesthesia) OR spinal block) OR regional anesthesia) OR subarachnoid block | 83040 |
| 18 | Search (((((((((((femoral nerve block) OR Fentanyl) OR morphine) OR tramadol) OR Nalbuphine) OR analgesic) OR analgesia) OR pain) OR position)) AND ((((spinal anesthesia) OR spinal block) OR regional anesthesia) OR subarachnoid block)) AND fracture | 1008 |
| Recent queries in EMBASE on January 15, 2018 | | |
| 1 | (((((((((((femoral nerve block) OR Fentanyl) OR morphine) OR tramadol) OR Nalbuphine) OR analgesic) OR analgesia) OR pain) OR position)) AND ((((spinal anesthesia) OR spinal block) OR regional anesthesia) OR subarachnoid block)) AND fracture | 1016 |
| Recent queries in Cochrane database on January 15, 2018 | | |
| 1 | (((((((((((femoral nerve block) OR Fentanyl) OR morphine) OR tramadol) OR Nalbuphine) OR analgesic) OR analgesia) OR pain) OR position)) AND ((((spinal anesthesia) OR spinal block) OR regional anesthesia) OR subarachnoid block)) AND fracture | 191 |
| Recent queries in Scopus on January 12, 2018 | | |
| 1 | (((((((((((femoral nerve block) OR Fentanyl) OR morphine) OR tramadol) OR Nalbuphine) OR analgesic) OR analgesia) OR pain) OR position)) AND ((((spinal anesthesia) OR spinal block) OR regional anesthesia) OR subarachnoid block)) AND fracture | 525 |
| Others | |  |
| 1 | searching references | 4 |
