# Supplementary material for: Efficacy and safety of femoral nerve block for the positioning of femur fracture patients before a spinal block – A systematic review and meta-analysis
Source: PLoS One. 2019 May 2;14(5):e0216337. doi: 10.1371/journal.pone.0216337 (PMC6497313; doi:10.1371/journal.pone.0216337)
Supplement: S2 Table — (DOCX) [file pone.0216337.s003.docx]

S2 Table. Baseline characteristics of the included studies

| Study | Age: years, mean (SD) | | Gender (% of male) | | Weight(kg) | | Time from trauma to surgery (days) | | ASA(I/II/III/IV), N | | Site of Femur Fracture (Proximal/shaft/distal), N | |
| --- | --- | --- | --- | --- | --- | --- | --- | --- | --- | --- | --- | --- |
|  | FNB | IVA | FNB | IVA | FNB | IVA | FNB | IVA | FNB | IVA | FNB | IVA |
| Sia 2004 | 35(11) | 32(9) | 70 | 60 | 71(11) | 69(10) | 2.5(1.7) | 2.3(2.1) | NA | NA | 10/0/0/0 | 10/0/0/0 |
| Szucs 2012 | 76.0(13.7) | 80.2(5.1) | 50 | 16 | NA | NA | NA | NA | NA | NA | NA | NA |
| Durrani 2013 | 40.79(16.9) | 44.12(17.8) | 60 | 71 | NA | NA | 4.69(3.30) | 5.40(4.31) | 26/10/6/0 | 26/6/10/0 | 22/16/04 | 25/12/05 |
| Jadon 2014 | 63.3(11.7) | 65.3(16.7) | 63 | 70 | 62.8(13.7) | 64.6(9.7) | NA | NA | 4/20/6/0 | 5/21/4/0 | 23/7/0 | 22/8/0 |
| Reddy 2016 | 62.4(9.4) | 65.1(6.8) | 60 | 60 | 67.1(12.1) | 63.8(8.3) | NA | NA | 3/29/4/0 | 5/24/7/0 | 24/5/7 | 28/3/5 |
| Ranjit 2016 | 61.8(16.8) | 61.65(15.8) | 75 | 50 | NA | NA | NA | NA | 13/7/0/0 | 1/19/0/0 | NA | NA |
| Vats 2016 | 47.5(14.4) | 45.4(15.5) | 74 | 72 | NA | NA | NA | NA | NA | NA | 37/12/1 | 36/13/1 |
| Iamaroon 2010 | 65.1(17.5) | 68.2(12.4) | 34 | 37 | 58.2(9.1) | 57.6(10.0) | 8.0(7.0) | 15.6(18.4) | 8/20/4/0 | 4/23/5 | 26/6/0 | 28/1/3 |
| Pakhare 2016 | NA | NA | NA | NA | NA | NA | NA | NA | NA | NA | NA | NA |
| Singh 2016 | 51.7(15.0) | 48.2(15.2) | 63 | 63 | NA | NA | NA | NA | 22/8/0/0 | 23/5/7/0 | 27/3/0 | 29/1/0 |

Footnotes: Data described as mean ± SD. Abbreviations: FNB, femoral nerve block; IVA, intravenous analgesic; NA, not available; ASA, American Society of Anesthesiologists; N, number
