# Supplementary figures and images for: Efficacy and safety of femoral nerve block for the positioning of femur fracture patients before a spinal block – A systematic review and meta-analysis
Source: PLoS One. 2019 May 2;14(5):e0216337. doi: 10.1371/journal.pone.0216337 (PMC6497313; doi:10.1371/journal.pone.0216337)

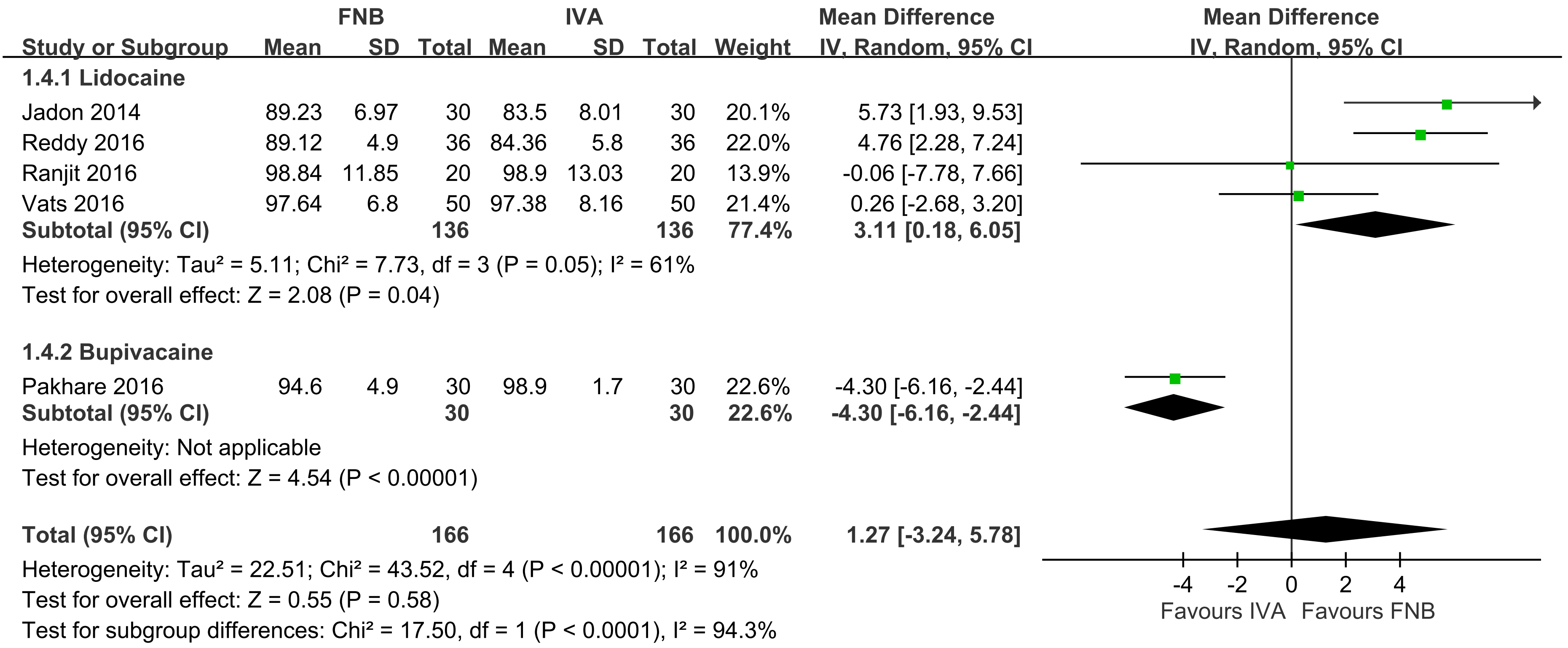

Supplement: S1 Fig — (TIF) [file pone.0216337.s004.tif]

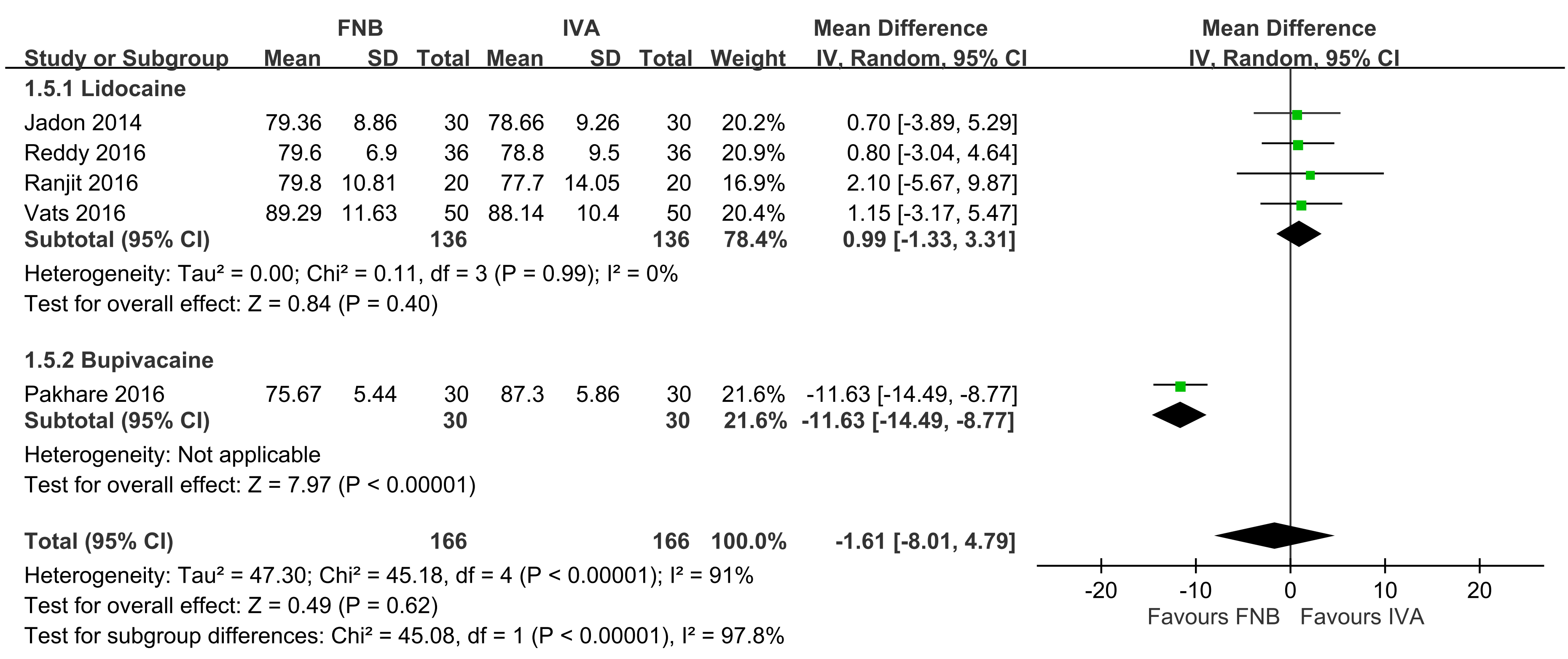

Supplement: S2 Fig — (TIF) [file pone.0216337.s005.tif]

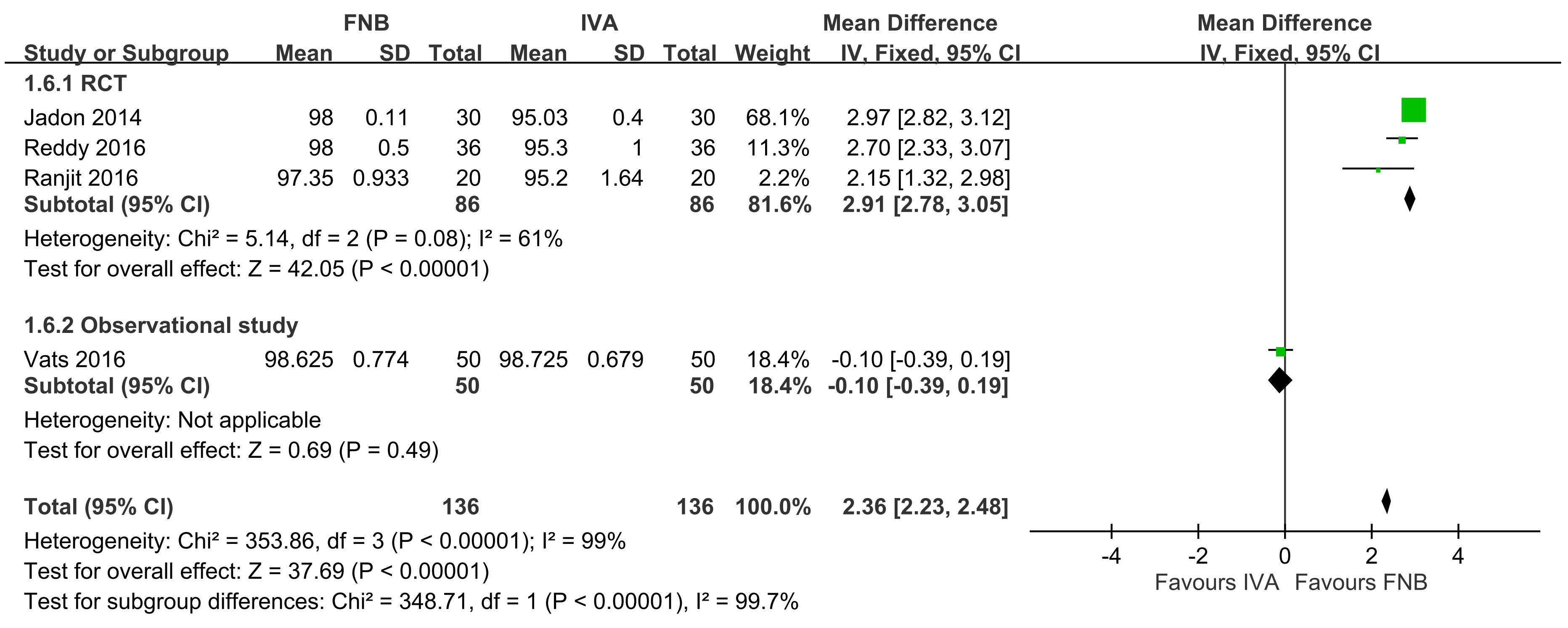

Supplement: S3 Fig — (TIF) [file pone.0216337.s006.tif]
